# Aboveground Biomass of Wetland Vegetation Under Climate Change in the Western Songnen Plain

**DOI:** 10.3389/fpls.2022.941689

**Published:** 2022-06-17

**Authors:** Yanji Wang, Xiangjin Shen, Shouzheng Tong, Mingye Zhang, Ming Jiang, Xianguo Lu

**Affiliations:** ^1^Northeast Institute of Geography and Agroecology, Chinese Academy of Sciences, Changchun, China; ^2^University of Chinese Academy of Sciences, Beijing, China

**Keywords:** marsh wetland, aboveground biomass, vegetation, climate, Songnen Plain

## Abstract

Understanding the spatiotemporal dynamics of aboveground biomass (AGB) is crucial for investigating the wetland ecosystem carbon cycle. In this paper, we explored the spatiotemporal change of aboveground biomass and its response to climate change in a marsh wetland of western Songen Plain by using field measured AGB data and vegetation index derived from MODIS datasets. The results showed that the AGB could be established by the power function between measured AGB density and the annual maximum NDVI (NDVI_max_) of marsh: Y = 302.06 × NDVI_max_^1.9817^. The averaged AGB of marshes showed a significant increase of 2.04 g⋅C/m^2^/a, with an average AGB value of about 111.01 g⋅C/m^2^ over the entire western Songnen Plain. For the influence of precipitation and temperature, we found that the annual mean temperature had a smaller effect on the distribution of marsh AGB than that of the total precipitation in the western Songnen Plain. Increased precipitation in summer and autumn would increase AGB by promoting marshes’ vegetation growth. In addition, we found that the minimum temperature (T_min_) and maximum temperatures (T_max_) have an asymmetric effect on marsh AGB on the western Songnen Plain: warming T_max_ has a significant impact on AGB of marsh vegetation, while warming at night can non-significantly increase the AGB of marsh wetland. This research is expected to provide theoretical guidance for the restoration, protection, and adaptive management of wetland vegetation in the western Songnen Plain.

## Introduction

Marsh is one of the most widely distributed wetland types and plays a vital role in climate regulation and the global carbon cycle (Shen et al., 2021a,b). Biomass is the main input of organic carbon in terrestrial ecosystems (Scurelock et al., 2002; Shen et al., 2020). The aboveground biomass (AGB) is a representative of primary production, which is an important indicator of ecosystem carbon stocks in wetlands (Flombaum and Sala, 2007; Chopping et al., 2008; Wang et al., 2021). Climate change can have a critical effect on marsh biomass, and thus impact regional carbon stocks (Shen et al., 2021a; Wang et al., 2021). In the past several years, numbers of studies on the response of AGB to climate have concentrated on other ecosystems (i.e., grassland and forest ecosystem) (Piao et al., 2004; Fang et al., 2010; Gao et al., 2013; Mitsch et al., 2013; Shen et al., 2021a, 2022a,b; Wang et al., 2021, 2022; Yang et al., 2021). With the rapid development of remote sensing, many studies have analyzed the correlations between MODIS data and biomass in different regions (Muukkonen and Heiskanen, 2007; Caccamo et al., 2011; Dong et al., 2016; Yin et al., 2019; Gao et al., 2020). Until recently, temporal-spatial changes in the AGB of marsh wetland ecosystems and their response to climate change at the regional scale have not been elucidated. To study the regional carbon cycle and sustainable use of marsh resources, it is important to comprehend the AGB dynamics of marshes and their relation to climate change (Byrd et al., 2018).

The western Songnen Plain, located in an ecologically fragile zone in Northeast China, which is expected to influence marsh growth and productivity, is highly sensitive to climate change (Xie et al., 2020). The area includes large marsh wetlands, which help to regulate the global carbon cycle (Wang et al., 2011, 2020; Shen et al., 2019, 2021b). Therefore, investigating the vegetation AGB changes and the relationship between vegetation AGB and climate change is important for assessing the carbon storage capacity in marsh wetland ecosystems in the western Songnen Plain. Previous studies have explored the changes of marsh vegetation biomass in the western Songnen Plain. For example, Yang and Li (2003) analyzed the biomass of *Phragmites communis* populations in the Songnen Plain, and Guan et al. (2018) studied the vegetation biomass in the Xianghai Wetland. However, most studies have focused on community scales or small study areas because of their limitations in field measurements. With the rapid progress of remote sensing, a combination of remote sensing and ground observations datasets at different scales can be used to effectively estimate vegetation biomass (Myneni et al., 1997, 2001; Piao et al., 2005, 2007; Fang et al., 2007). For instance, Piao et al. (2003) estimated the magnitude and changes in grassland biomass in some ecosystems in China. Yang et al. (2010) investigated the AGB of Tibetan grasslands. Gao et al. (2013) estimated the AGB of Inner Mongolia’s grasslands. Several previous studies have also investigated the spatiotemporal dynamic patterns of AGB in the western Songnen marsh Plain by combining remote sensing data and field observation data. To accurately clarify the spatiotemporal change in marsh AGB and its response to climate change on the western Songnen Plain, it is useful to estimate the marsh AGB using large-scale remote-sensing and field observation datasets. Under the background of global warming, previous researchers have found that minimum temperatures at night have increased more rapidly in the past than maximum temperatures during the day (Peng et al., 2013; Shen et al., 2014). This asymmetric warming pattern is more likely to occur during the day and at night (Peng et al., 2013; Qiao et al., 2014). Some studies found that the influences of day and night temperatures on vegetation growth are different. For example, Shen et al. (2021b) found that an increase in minimum temperature significantly promoted the growth of marsh vegetation. Wang et al. (2022) discovered that the increase of maximum temperature has a negative effect on the marsh vegetation growth. At present, whether warming maximum and minimum temperatures had asymmetric impacts on AGB of marsh vegetation in western Songnen Plain is unclear. Therefore, in the context of global asymmetric day and night warming, it is very important to investigate the influences of daytime maximum temperature and night minimum temperature on AGB of marsh vegetation in the western Songnen Plain.

In the present study, we established an evaluation model of marsh AGB using measured AGB data from the western Songnen Plain marshes and the annual NDVI_max_. Subsequently, we explored the spatiotemporal variation in AGB of marsh vegetation and its relationship with climate factors [including precipitation, mean temperature (T_mean_), maximum temperature (T_max_), and minimum temperature (T_min_)]. The objective of this study was to describe the spatiotemporal changes of marshes AGB and their response to climate change, which is crucial for the restoration and protection of wetland plants.

## Materials and Methods

### Study Area

**FIGURE 1:**
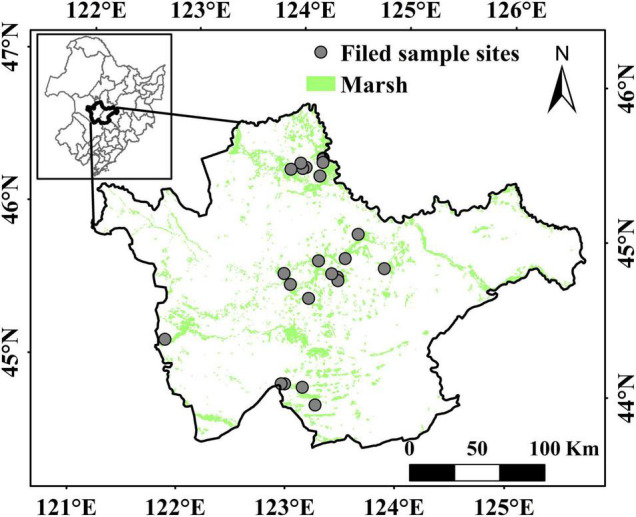
Spatial distribution of marsh and aboveground biomass filed sampling points on the western Songnen Plain.

The western Songnen Plain (43°59′–46°18′N, 121°83′–126°30′E) is located in the west of Jilin Province and covers a total area of 46.9 × 10^3^ km^2^ (Figure 1). The study area is a semi-arid continental monsoon climate zone characterized by four seasons: windy springs, rainy summers, windy autumns, and cold winters. The mean annual temperature spatially increases from north to south (4–6°C). The precipitation is between 350 and 650 mm, 70–80% of which is concentrated in June–August, and gradually decreases from east to west (Wang et al., 2020). The northern edge of the study area includes the Songhua River, the Nenjiang River, and a few river branches (Wang et al., 2009). The vegetation of the area is composed of typical marsh plants distributed in wetlands and mainly includes *Deyeuxia angustifolia*, *Typha orientalis, Bolboschoenus planiculmis*, and *Phragmites australis* (Liu et al., 2018; Zhang et al., 2021).

### Data Analysis

Data on monthly climate during 2000–2020 were created from meteorological stations in the western Songnen plain and were provided by the National Meteorological Information Center.^1^ We adopted the ordinary Kriging method to interpolate the meteorological station data into the marsh distribution in western Songnen Plain by using ARCGIS software. The spatial resolution and projected coordinates of the interpolated climate data were unified into the same as the MODIS data information. The MOD13Q1 NDVI dataset for the period from 2000 to 2020 was acquired from the National Aeronautics and Space Administration (NASA), United States. Data for this period with spatial and temporal resolutions of 250 m and 16 days, respectively, were used for the dynamic change analysis. To obtain the maximum annual NDVI (NDVI_max_) values, the maximum value composite (MVC) was used to reconstruct the 16-day NDVI into NDVI_max_ values (Holben, 1986; Stow et al., 2003; Pettorelli et al., 2005; Zhang et al., 2012; Wang et al., 2020). In addition, to obtain the unaltered marshes of the western Songnen Plain, two marsh maps for the year 2000 and 2015 covering the western Songnen Plain were also used in this study (Mao et al., 2019). These marsh wetland distribution data with a spatial resolution of 30 m × 30 m was taken from the National Geographic Resource Science Sub-Center of China.^2^ The ground truth points used for verification of the overall accuracy of marsh datasets are generally accurate (Mao et al., 2019). The field biomass sampling survey was collected from July to September of 2012–2016 in the western Songnen plain. In the field survey, we used the AGB data of 24 marsh sites over the entire study area by adopting a comprehensive sampling method to acquire more accurate AGB values (Figure 1). In addition, a total of 24 field sample sites in the marsh distribution patches were selected based on the distribution of main marsh patches, and three repeat quadrats of equal size (1 m × 1 m) were set for each site (Shen et al., 2021a). When selecting repeated quadrats, the investigators fully considered the average status of vegetation in the marsh patches. Because the average AGB density of each field site was calculated from the mean AGB density of three quadrats in the same site, the AGB density could basically reflect the mean aboveground biomass density at each field site in the marsh patches. According to a previous study, a conversion coefficient of 0.45 can be used to convert the aboveground biomass values to carbon contents (Wang et al., 2021).

### Methods

#### Analysis of Aboveground Biomass Dynamics

Piao et al. (2004) showed that there is a correlation between the NDVI_max_ and the AGB of grasslands in China. Based on previous studies, we established the relationship between the marsh AGB data from filed-observation at 24 survey sites and the corresponding NDVI_max_ values to estimate the AGB of marsh vegetation at each pixel in western Songnen Plain in the period from 2000 to 2020. For the AGB calculation, we extracted the NDVI_max_ value of each sampling site from the NDVI_max_ according to the corresponding geographic location (Ding et al., 2007).

#### Evaluation and Analysis of the Modeled Estimates

We calculated the coefficient of determination (*R*^2^) and the Root Mean Square Error (RMSE) to compare the predicted values with observations (Piao et al., 2007).

$$R^{2}=1-\frac{\sum_{\text{n}=1}^{\text{x}}{\left({\text{x}}_{\text{obs}}-{\text{x}}_{\text{est}}\right)}^{2}}{\sum_{\text{n}=1}^{\text{x}}{\left({\text{x}}_{\text{obs}}-\bar{{\text{x}}_{\text{est}}}\right)}^{2}}$$

$$\text{RMSE}=\sqrt{\frac{\sum_{\text{n}=1}^{\text{x}}{\left({\text{x}}_{\text{est}}-{\text{x}}_{\text{obs}}\right)}^{2}}{\text{x}}}$$

where x_*est*_ is the estimated value of the AGB, x_*obs*_ is the observed value of the AGB, $\bar{x_{\text{est}}}$ is the average of the estimated value, and x is the number of samples (24). The RMSE is a measure of prediction accuracy.

#### Trend Analysis

This study used the simple liner regression to calculate the trends of marsh NPP and climate variables from 2000 to 2020. The calculation formula is as follows (Wang et al., 2020):

$$Slope=\frac{n*\sum_{i=1}^{n}i*AGB_{i}-(\sum_{i=1}^{n}i)(\sum_{i=1}^{n}AGB_{i})}{n*\sum_{i=1}^{n}i^{2}-{\left(\sum_{i=1}^{n}i\right)}^{2}}$$

*n* is the number of years analyzed; *AGB*_*i*_ is AGB during the *i* year; *Slope* is the trend of NPP or climate variables for each pixel. If the *Slope* < 0 (> 0), it means a decrease (an increase) of the AGB during 2000–2020; when the *Slope* is zero, it shows that the AGB has no significant change during 2000–2020.

## Results

### Estimation and Verification of Marsh Aboveground Biomass in the Western Songnen Plain

**FIGURE 2:**
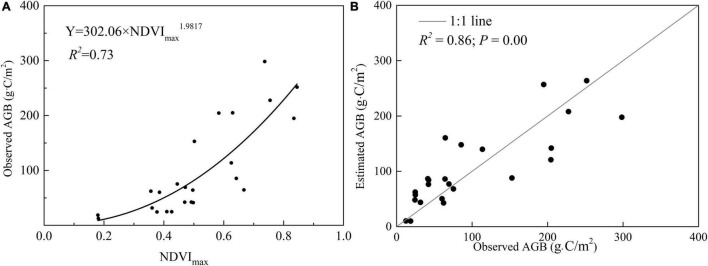
Equation between observed AGB and NDVI_max_ of marsh vegetation **(A)** and relationship between observed AGB and estimated AGB **(B)** in the marsh of western Songnen Plain.

**FIGURE 3:**
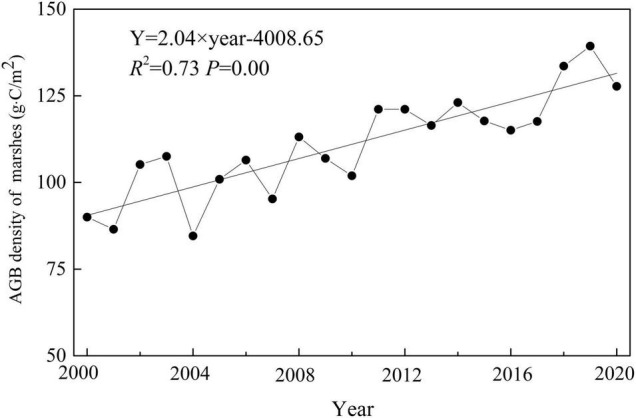
Temporal variation of aboveground biomass density on the western Songnen Plain marshes from 2000 to 2020.

Regression models (power function) for each paired NDVI_max_ and field-measured AGB of marsh vegetation were estimated (Figure 2). The model was evaluated by calculating the RMSE and *R*^2^, and the observed values were compared with the estimates. The RMSE and *R*^2^ were 42.60 and 0.98, respectively. Based on Y = 302.06 × NDVI_max_^1.9817^ (*R*^2^ = 0.73) the average AGB density of marshes over the entire western Songnen Plain from 2000 to 2020 was estimated (Figure 3). Overall, the average estimated marsh AGB was approximately 111.01 g⋅C/m^2^. The *R*^2^ value between the observed and estimated AGB was 0.86, being extremely significant (*P* < 0.01).

### Spatiotemporal Variations of Aboveground Biomass in the Western Songnen Plain Marsh Wetland

**FIGURE 4:**
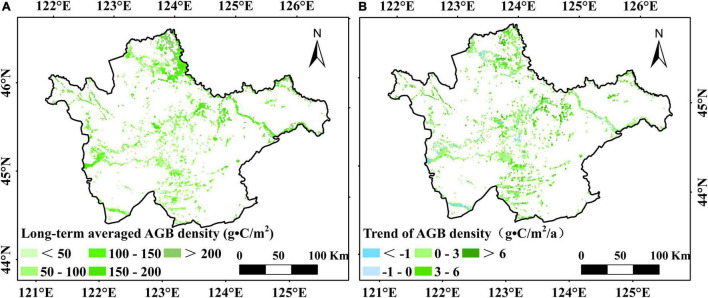
Spatial patterns of the long-term averaged AGB density **(A)** and change trend of AGB density **(B)** in the western Songnen Plain marshes during 2000–2020.

The temporal changes in marsh AGB density in the western Songnen Plain are shown in Figure 3. This study discovered that the AGB density value of marsh vegetation over the entire western Songnen Plain had a significant increase of 2.04 g⋅C/m^2^/a during the study period of 2000–2020 (Figure 3). Higher AGB appeared in the northern part of the western Songnen Plain marsh wetland (Momoge Nature Reserve) when compared to the southern part (Figure 4A). During the past two decades, the annual trend in AGB of marsh vegetation on the western Songnen Plain showed distinct-obvious spatial heterogeneities; the decreasing marsh AGB trend was mainly observed in the central western Songnen Plain (Figure 4B).

### Impact of Climate Variables on the Aboveground Biomass of Wetland Vegetation

**FIGURE 5:**
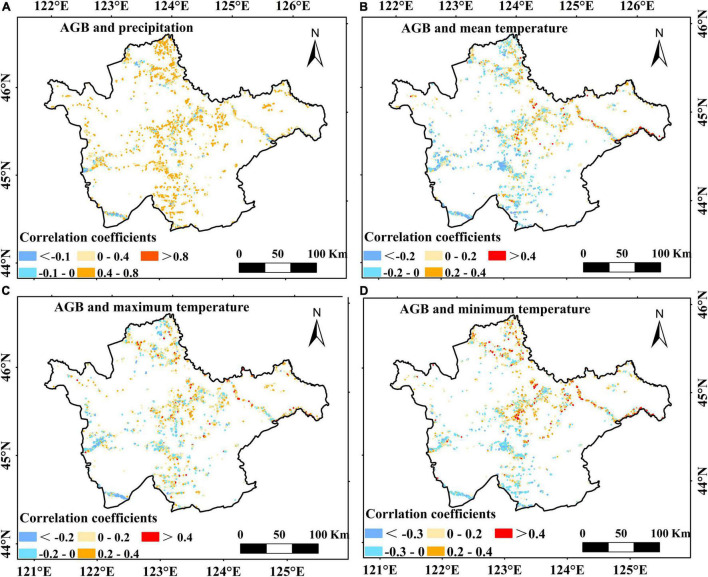
Spatial distributions of correlation coefficients between the annual AGB and annual precipitation **(A)**, annual mean temperature **(B)**, annual maximum temperature **(C)**, and annual minimum temperature **(D)** in the western Songnen Plain marshes.

Figure 5 shows the response of spatial distribution of AGB in the investigated area to annual precipitation and temperature (T_mean_, T_max_, and T_min_) over the western Songnen Plain wetland. We found that the correlation between AGB and climatic factors showed spatial heterogeneity (Figure 5B). Moreover, there was a significant positive correlation between marsh AGB and total precipitation (*P* < 0.01), and a weak correlation with T_mean_ (Table 1 and Figures 5A,B). Regarding the maximum and minimum temperatures, our results found that the correlation between marsh AGB and T_min_ was lower than that between marsh AGB and T_max_ (Table 1 and Figures 5C,D). The correlation between AGB and T_max_ was significantly positive (*P* < 0.05) over the study area.

**TABLE 1:** Correlations between climate factors and AGB of marsh vegetation in the western Songnen Plain marshes.

	Precipitation	T_mean_	T_max_	T_min_
Annual	0.746**	0.316	0.451*	0.166
Spring	0.214	0.178	0.489*	0.400
Summer	0.691**	0.153	0.129	0.303
Autumn	0.446*	–0.041	–0.066	0.225
Winter	0.070	0.114	0.256	0.189

Levels of significance are set at *p < 0.05 and **p < 0.01.

Regarding the impact of seasonal precipitation on marsh AGB, the correlation between aboveground biomass and precipitation during the summer and autumn periods was significantly positive (Table 1), indicating that an increasing total precipitation would increase biomass by promoting marsh vegetation growth during the summer and autumn periods. Regarding the influence of T_max_ and T_min_, marsh AGB has a significant positive correlation with T_max_ in spring (Table 1). By contrast, a weak positive relationship was observed between marsh AGB and T_min_ in all seasons (Table 1).

## Discussion

### Estimation of the Aboveground Biomass in the Western Songnen Plain Wetland

In the present study, we established the fitting equation between marsh AGB and NDVI_max_ for each pixel. The power equation was Y = 302.06 × NDVI_max_^1.9817^ (Figure 2A; *R*^2^ = 0.73). Our findings were similar to those of Piao et al. (2004), who discovered that the grassland aboveground biomass and the NDVI_max_ value had a better power function. However, there are some differences between these functions. Piao et al. (2004) explored AGB of the grassland of China, whereas the present study explored the AGB of temperate semi-arid and semi-humid marshes in the western Songnen Plain. In the present study, the RMSE and *R*^2^-values of the fitted equations were 42.60 and 0.98, respectively. These values indicated that the estimating equation could correctly calculate the marsh AGB on the western Songnen Plain. Furthermore, the regression models established between NDVI_max_ and field-measured AGB values in the marsh provided a new method for investigating wetland AGB in the western Songnen Plain.

Based on this equation and NDVI_max_ datasets, we calculated the average AGB density of the studied marsh wetland over a period of 20 years. The results revealed higher marsh AGB in the north of the western Songnen Plain (Momoge Nature Reserve) than in the southern part (Figure 4A). The reason for this may be that the abundant precipitation creates favorable environmental conditions for the growth of marsh vegetation in this region. The average marsh AGB showed a significant increasing trend (2.04 g⋅C/m^2^/a) over the years, with an average AGB density of about 111.01 g⋅C/m^2^ over the entire western Songnen Plain.

### Correlations Between Climate Variables and Vegetation Aboveground Biomass on the Western Songnen Plain Wetland

Multiyear AGB values and the corresponding climate dataset from 2000 to 2020 were used to analyze the annual AGB trends and their relationships with seasonal climate. These trends suggested large spatiotemporal heterogeneity, mainly corresponding to the seasonal changes of climate, indicating that seasonal climate change plays a crucial role in AGB trends. The correlations between marsh AGB and precipitation, T_mean_, T_max_, and T_min_ were studied, and the results are shown in Figure 5.

In the present study, marsh AGB had a significant positive correlation with total precipitation, especially during the summer and autumn, but it was weakly positively correlated with the mean temperature. Previous studies have shown that the AGB of marsh vegetation in the studied area is less sensitive to mean temperature than to precipitation (Shen et al., 2019; Wang et al., 2020, 2022). Our findings confirmed that increasing precipitation in summer and autumn could be beneficial for marsh vegetation growth on the western Songnen Plain. This result is similar to that obtained by Wang et al. (2013), who discovered that during the growing season on the western Songnen Plain, the NDVI of marsh vegetation has a significant positive relationship with the total precipitation. Relatively good hydrothermal conditions are favorable for wetland vegetation growth in the summer and autumn (Li et al., 2019). Increasing precipitation in these seasons might improve plant light use efficiency, leading to an increase in the AGB of marsh vegetation. In addition, we note that marsh distribution dataset used in this study could contain some seasonal marshes. Precipitation can change the marsh distribution in rainy season. Increasing precipitation in summer and autumn could increase the actual distribution area of marshes and increase moisture availability for marsh vegetation growth, thus increasing marsh aboveground biomass within a certain area (Wang et al., 2021). This may partially account for the positive impacts of precipitation on the AGB in the western Songnen Plain wetland.

Regarding the impact of temperature on marsh AGB, the AGB was positively and significantly correlated with T_max_ but had a weak positive correlation with T_min_. Our results further showed that T_min_ and T_max_ had an asymmetric effect on the aboveground biomass of the investigated marsh wetland (Table 1), suggesting that the increased maximum temperature in spring and winter may be associated with the temporal change in marsh AGB, especially during spring. Furthermore, our findings were consistent with previous studies showing that a warmer climate could lead to increase aboveground biomass as a consequence of enhanced photosynthetic rate and longer growth season (Myneni et al., 1997, 2001; Los et al., 2001; Tucker et al., 2001; Zhou et al., 2001; Hicke et al., 2002; Slayback et al., 2003). First, warming during the day accelerates the reaction process of the photosynthetic enzymes, thereby increasing its activity of the photosynthetic enzyme and being conducive to the accumulation of organic matter (Shen et al., 2016). Second, prolonged growing period and early spring phenology may promote carbon sequestration by vegetation (White et al., 1999; Piao et al., 2003). In addition, we found that the minimum temperature showed a significant increase compared to that in maximum temperature but may exert only a slight impact on vegetation growth. Studies have shown that warming at night can be beneficial for vegetation growth as it reduces frost (Shen et al., 2019). Therefore, all these factors may explain why increasing daytime and nighttime temperatures can accelerate the vegetation growth on the western Songnen Plain marsh wetland.

### Vegetation Changes in the Western Songnen Plain Marsh Wetlands

**FIGURE 6:**
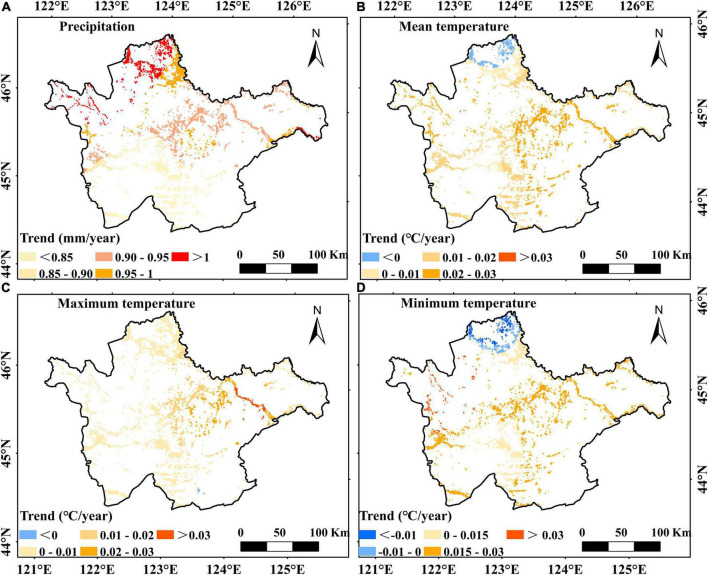
Spatial change trends of annual precipitation **(A)**, annual mean temperature **(B)**, annual maximum temperature **(C)**, and annual minimum temperature **(D)** in the western Songnen Plain marshes during 2000–2020.

In order to further investigate the variation in the AGB of the marshes, we analyzed the spatial-temporal changes in climate factors during the period from 2000 to 2020 (Table 2 and Figure 6). Precipitation showed a significantly increasing trend, at a rate of the 0.919 mm/a (*P* < 0.05), but no significant variation in T_mean_, T_max_, and T_min_ was observed. Moreover, precipitation was high in summer and autumn (1.378 mm/a and 1.334 mm/a, respectively) during the entire study period. Based on the correlations between marsh AGB and precipitation (Figure 4 and Table 1), we concluded that significant increases in summer and autumn precipitation may have accounted for the increasing marsh AGB. Increased rainfall in summer and autumn can enhance vegetation growth, as discussed in section “Correlations Between Climate Variables and Vegetation Aboveground Biomass on the Western Songnen Plain Wetland.” Regarding the temperature changes, the trends of T_min_ were positive in all seasons, suggesting that the T_min_ increased for all seasons during the study period (Table 2). In particular, summer T_min_ exhibited an increasing trend (0.087°C/a, *P* < 0.05). This significant summer T_min_ trend in the as well as the one in precipitation during summer and autumn may be beneficial to the increase in marsh AGB throughout the western Songnen Plain.

**TABLE 2:** Temporal trends of precipitation (mm/a), T_mean_ (°C/a), T_max_ (°C/a), and T_min_ (°C/a) on the western Songnen Plain marshes from 2000 to 2020.

	Precipitation	T_mean_	T_max_	T_min_
Annual	0.919**	0.015	0.021	0.013
Spring	0.877	–0.004	0.046	0.088
Summer	1.378*	–0.003	–0.022	0.087*
Autumn	1.334**	0.033	0.001	0.093
Winter	0.088	0.053	0.057	0.130

Levels of significance are set at *p < 0.05 and **p < 0.01. The symbol “a” indicates per year.

Regarding the spatial changes in the AGB, the greatest increase of marsh AGB during the study period was observed in the northern Songnen Plain (Figure 4B). In particular, our results found that this region had the largest increase in precipitation (Figure 6A). According to the spatial correlation between marsh vegetation AGB and total precipitation (Figure 5A), the significant increase of total precipitation in the same area may account for the increase in AGB of marsh vegetation in the northern Songnen Plain. In the past 20 years, the local government has strengthened wetland protection and management to restore marsh wetlands in the western Songnen Plain (Wang et al., 2022), which may partly explain the increased AGB of marsh vegetation in this study.

### Study Limitations

This study has some certain limitations. The first is the uncertainty of marsh data, which may have included some seasonal marshes; therefore, differences from the actual marsh distribution dynamics may lead to some uncertainties in the descriptions of the response of AGB to climate change. For the field-observation data, the ground points were limited and not uniformly distributed, which could cause some uncertainty about the results of this study. Further studies are still needed to verify our results using more field-observation sites. **The** second is the uncertainty of the NDVI time series dataset. In addition, the actual condition of vegetation in a certain area cannot be fully reflected by NDVI data. Third, owing to technical limitations, marsh AGB values cannot be accurately measured under the saturation condition of NDVI_max_. Fourth, we only discussed the impact of precipitation, T_mean_, T_max_, and T_min_ on the AGB of marsh vegetation in our studies. In order to reveal the mechanism of marsh AGB variation and simulate biomass results, the influences of various environmental factors on AGB need to be further investigated in the western Songnen Plain marshes in the future.

## Conclusion

We accurately estimated a power function (Y = 302.06 × NDVI_max_^1.9817^), and the aboveground biomass of marsh vegetation on the western Songnen Plain can be well estimated by using the remote sensing and measured biomass datasets. The marsh AGB density showed a significant increasing trend (2.04 g C/m^2^/a), with an average AGB density value of 111.01 g⋅C/m^2^ during the period 2000–2020 over the entire western Songnen Plain. An especially high AGB value was estimated for the north of the western Songnen Plain marsh wetland (Momoge Nature Reserve). Regarding the influence of precipitation and temperature, the AGB of marsh vegetation is less sensitive to temperature than to precipitation in this region. Increasing precipitation in summer and autumn would increase AGB values by promoting marshes vegetation growth. In addition, we found that the T_min_ and T_max_ have an asymmetric effect on AGB in the western Songnen Plain marsh wetland, with the maximum temperature warming significantly stimulating the vegetation growth. On the other hand, the minimum temperature showed a significant increase but may exert a slight impact on vegetation growth. The findings of the study can affect the protection and management of marshy regions in the future.

## Data Availability Statement

The original contributions presented in the study are included in the article/supplementary material, further inquiries can be directed to the corresponding author/s.

## Author Contributions

ST coordinated the project. YW carried out the data analysis and wrote the manuscript. XS, MZ, MJ, and XL contributed to modifying the manuscript. All authors contributed to the article and approved the submitted version.

## Conflict of Interest

The authors declare that the research was conducted in the absence of any commercial or financial relationships that could be construed as a potential conflict of interest.

## Publisher’s Note

All claims expressed in this article are solely those of the authors and do not necessarily represent those of their affiliated organizations, or those of the publisher, the editors and the reviewers. Any product that may be evaluated in this article, or claim that may be made by its manufacturer, is not guaranteed or endorsed by the publisher.

## Footnotes

1
http://www.nmic.cn/

2
http://gre.geodata.cn
